# *Epidermophyton floccosum*, an etiological agent of tinea pedis and tinea unguium: about two cases

**DOI:** 10.11604/pamj.2025.50.102.40580

**Published:** 2025-04-11

**Authors:** Rania Besrour, Latifa Mtibaa, Faten Rabhi, Nawel Baccouchi, Abderraouf Dhaoui, Boutheina Jemli

**Affiliations:** 1 Laboratory of Parasitology-Mycology, Military Hospital of Tunis, Tunis, Tunisia,; 2 Faculty of Medicine of Tunis, University of Tunis El Manar, Tunis, Tunisia,; 3 Department of Dermatology, Military Hospital of Tunis, Tunis, Tunisia,; 4 Faculty of Pharmacy of Monastir, Monastir, Tunisia

**Keywords:** *Epidermophyton floccosum*, dermatophyte, real-time PCR, tinea unguium, case report

## Abstract

Dermatophytia is an infection caused by keratinophilic filamentous fungi. The distribution of dermatophytes varies by country and geographic area. Epidermophyton (E) floccosum has experienced a downward trend in recent years. It is an anthropophilic dermatophyte that causes mainly skin infections in humans. We report two observations of a tinea pedis and a tinea unguium due to E. floccosum. A 70-year-old diabetic patient was admitted to dermatology to manage leg erysipelas. The patient had scaly plantar keratoderma and intertrigo of the toe-web spaces. Mycological sampling showed at direct examination mycelial filaments. A real-time Polymerase Chain reaction (PCR) allowed the identification of E. floccosum. The patient was treated with terbinafine ointment and short-term antibiotic therapy with favorable clinical course. A 70-year-old patient presented with subungual hyperkeratosis of the two big toenails, longitudinal melanonychia on the right, xanthonychia and distal onycholysis on the left. Microscopic examination of fungal colonies allowed the identification of Epidermophyton floccosum. A confirmation of the species diagnosis was carried out by real-time PCR. The patient was treated with terbinafine 250 mg daily for 6 months. Conventional techniques are, in most cases, sufficient for diagnosing superficial mycosis. However, identification is sometimes difficult, hence the importance of the contribution of molecular biology in the diagnosis.

## Introduction

Dermatophytia is an infection caused by keratinophilic filamentous fungi that can invade and infect keratinized tissues of humans and some animals. Seven fungal genera are described in the classification of De Hoog *et al*.: *Arthroderma, Epidermophyton, Lophophyton, Microsporum, Nannizia, Paraphyton, and Trichophyton* [1]. Depending on the source of the keratin used, dermatophytes can be classified into geophilic, zoophilic, and anthropophilic [1]. Several studies have shown that the epidemiology of dermatophytes has changed in recent years. The rate of isolation of *E. floccosum* compared to other dermatophytes has decreased gradually since the 1980s until the 2000s; the most common sites affected by this genus were skin and nails [2]. *E. floccosum* represented only 0.4-2.8% of isolates. It was isolated in 0.7% of cases of dermatophytosis and was most frequently present in intertrigo [2,3]. The exact reasons for such changes are not yet clearly defined. For some authors, it seems that the distribution of dermatophytes varies between countries and geographical areas and depends on several factors, such as lifestyle, migration of people, and climatic conditions. We report two rare observations of tinea pedis and a tinea unguium due to *E. floccosum* in elderly patients.

## Patient and observation

### Case 1

**Patient information:** a 70-year-old patient with history of diabetes type 2 on oral antidiabetics and rheumatoid arthritis on tocilizumab, presented with fever and painful swelling of the right leg.

**Clinical findings:** clinical examination showed erythematous, well-demarcated, warm and tender to the touch plaque of the leg, scaly plantar keratoderma and intertrigo of all toe web spaces.

**Figure 1:**
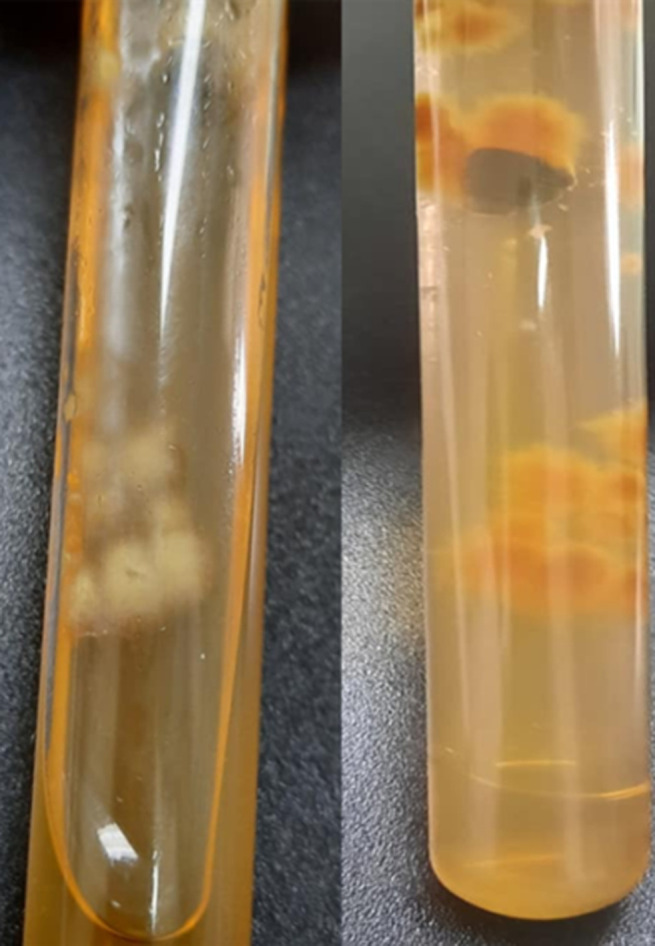
macroscopic morphology of the culture on Sabouraud medium (surface and reverse) showing radiated powdery colonies of khaki yellow color

**Figure 2:**
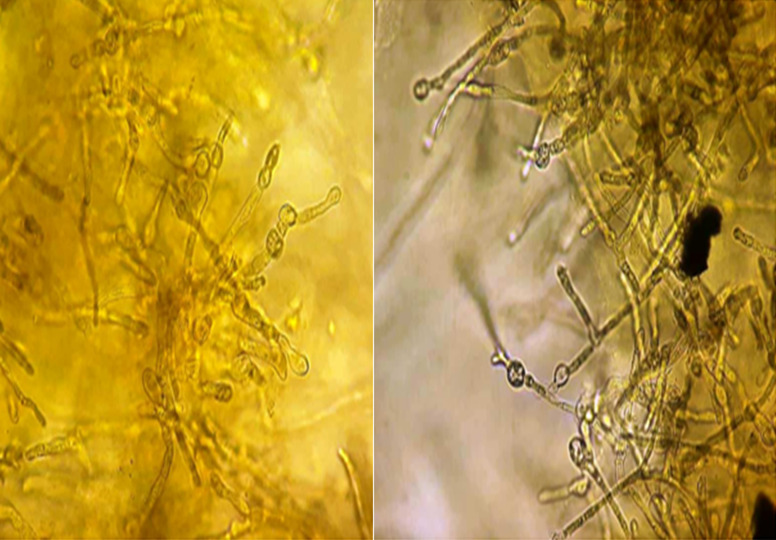
direct examination of the colonies showing terminal and intercalary chlamydospores (x40)

**Figure 3:**
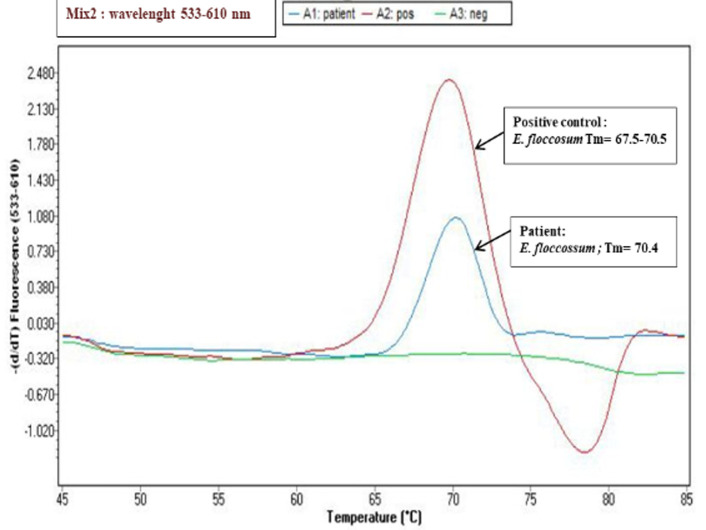
the melting curves seen on the LightCycler 480II with the real-time PCR (DermaGenius® 2.0); identification of *E. flocossum*

**Diagnostic assessment:** a mycological sampling on the Toe web spaces and soles of feet was carried out. Direct examination objectified mycelial filaments. The culture after 5 days showed radiated powdery colonies of khaki yellow color (Figure 1). Direct examination of the colony showed terminal and intercalary chlamydospores (Figure 2). Faced with the difficulty of morphological identification, a real-time PCR was performed using the DermaGenius® 2.0 kit (PathoNostics, the Netherlands). Data analysis was performed using the 2^nd^-derivative and Tm-calling function of the LC480 software (version 1.5.1.62 SP2). This allowed the identification of *Epidermophyton floccosum* (Figure 3).

**Therapeutic interventions:** the patient was put on amoxicillin and clavulanic acid and terbinafine ointment twice daily.

### Case 2

**Figure 4:**
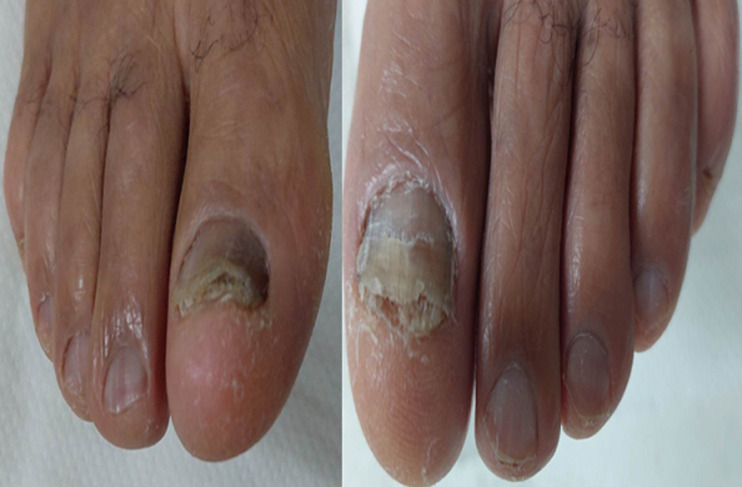
clinical aspect of lesions of both big toes

**Patient information:** a 70-year-old patient with no medical history consulted for damage to the nails of the two big toes that had been evolving for one year (Figure 4).

**Clinical findings:** clinical examination showed bilateral subungual hyperkeratosis, longitudinal melanonychia on the right, xanthonychia and distal onycholysis on the left.

**Figure 5:**
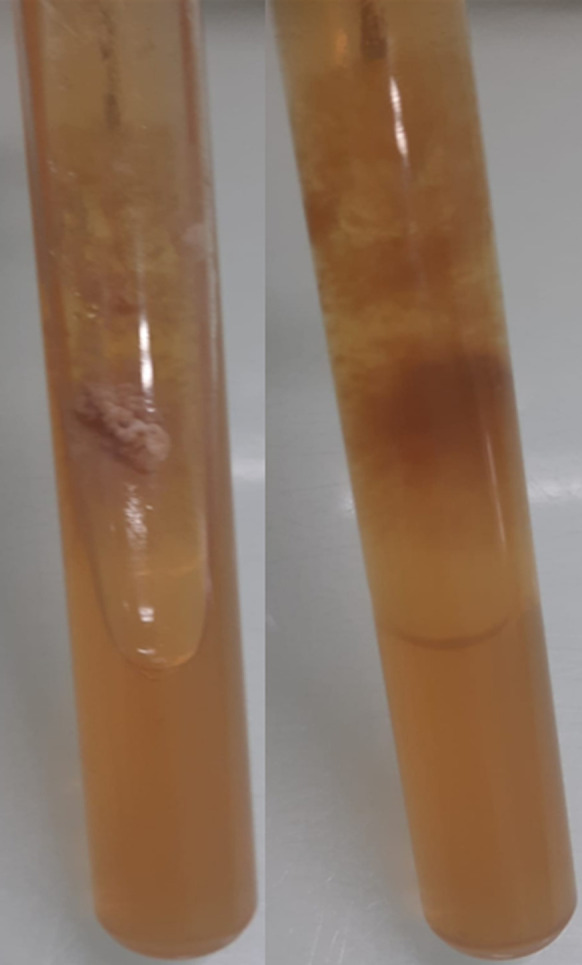
macroscopic morphology of the culture of on Sabouraud medium (surface and reverse) showing beige glabrous colonies

**Figure 6:**
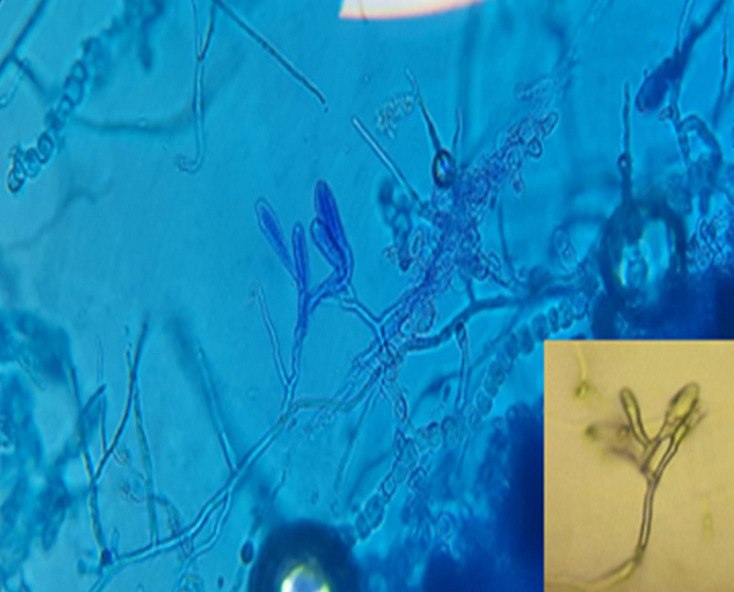
microscopic examination of the colonies after culture on agar water showing arthrosporous mycelial filaments, chlamydospores, and macroconidia clustered in a banana bunch (x40)

**Diagnostic assessment:** a mycological sample was taken with visualization at direct examination of mycelial filaments. The culture showed beige glabrous colonies on the front and smooth brown on the back (Figure 5). The colonies were sub-cultured on water agar for 5 days. Microscopy examination showed intercalary and terminal chlamydospores with club-shaped macroconidia clustered in a banana bunch (Figure 6). *Epidermophyton floccosum* was identified on these microscopic criteria as well as confirmation by real-time PCR (same PCR as that of the first case).

**Therapeutic interventions:** the patient was put on terbinafine 250 mg daily for 6 months (patient ongoing treatment).

**Informed consent:** it was obtained from both patients involved in the study.

## Discussion

The identification of dermatophytes is based mainly on macroscopic and microscopic morphological criteria of the colonies. In addition, morphological similarities between different species and/or polymorphism of some species are increasingly observed. Thus, identification of the isolated dermatophyte in culture can be difficult (case 1). Traditional laboratory methods for identification of dermatophytes are slow or non-specific, so there is a need for improved diagnostic methods. The application of nucleic acid amplification technology has allowed rapid and accurate identification of dermatophytes, and molecular biology approaches based on genetic markers have recently been very successful. Our study has shown the contribution of molecular biology in the early diagnosis of species with high sensitivity and specificity of the real-time PCR technique. Dermagenius® 2.0 kit allows DNA to be obtained from culture and even from the sample, whatever nails, skin or hair. In addition, results of mycological diagnosis require a month to isolate and identify of dermatophyte involved. Although the use of real-time PCR in routine technique is very expensive especially since the number of patients who consult for dermatophytosis is very high.

*Epidemophyton floccosum* is an anthropophilic species whose frequency has decreased compared to other dermatophyte species. In Chile, Cruz *et al*. reported that the *E. floccosum* isolation rate fell from 1.05% in 1980 to 0.21% in 2000 [2]. An additional study conducted in Southwestern Poland between 2011 and 2016 showed that *E. floccosum* represented only 1.9% of isolates [3]. However, a study of 1548 cases of dermatophytosis in Iran between 2003 and 2012 showed that *E. floccosum* was the third most commonly isolated species (13%), following *T. verrucosum* (40.6%) and *T. mentagrophytes* var. *Interdigitale* (17.6%) [4]. In the two cases we report, the patients were male and 70 years old. Dermatomycosis and dermatophytosis have been reported to be more common in women [4]. However, *E. floccosum* seems to be more common in men. Indeed, a study in Chile showed that *E. floccosum* infections predominated in men [2]. In a study by Park *et al*., which included 57 patients, the male to female ratio was 10.4 (52 men/5 women), and most infections occurred in the 10-29-year age group [5]. Cruz *et al*. in a study of 26 *E. flocossum* isolates, found that the most affected group was male patients in the age group of 36-60 years old (12.46%). Regarding age, studies agree that the infections occur mainly in the 30-60-year-old age group [2].

We report two observations of a tinea pedis and a tinea unguium due to *E. floccosum*. This agent frequently causes tinea cruris, tinea pedis, tinea corporis but not tinea capitis. The sites most frequently affected by this agent according to several surveys are essentially the intertrigo and the soles of the feet [2]. However, several authors reported rare cases of tinea capitis due to this fungus. Gawdzik *et al*.'s study on the epidemiology of dermatophytoses in the pediatric population in southwestern Poland between 2011 and 2016 reported a case of tinea capitis in a child caused by *E. floccosum* [3]. Chandra *et al*. reported a case of tinea capitis caused by *E. floccosum* in a 15-year-old girl [6]. *E. floccosum* is less and less implicated in human pathology. It rarely causes onyxis of the toenails, generally following a neglected intertrigo. Indeed, a study conducted in Iran between 2003 and 2012 on 625 cases of onychomycosis, *E. floccosum* accounted for only 0.7% of isolates [7].

The morphological similarities between different species and/or polymorphism of some species are increasingly observed. Thus, identification of the isolated dermatophyte in culture can be difficult. Recently, several novel molecular techniques have been developed for the rapid and accurate identification of pathogenic dermatophytes, conventional PCR with sequencing, and PCR-RFLP for analysis of the internal transcribed spacer (ITS) region [8]. In 2016, De Hoog *et al*. performed phylogenetic analysis of 261 dermatophyte reference strains, based on sequencing of five loci. The results indicate that the ITS sequence is sufficiently informative for genomic analyses of dermatophytes. The phylogenetic study of De Hoog has proposed significant changes in the taxonomy of dermatophytes. Seven main clades can be distinguished: *Arthroderma, Epidermophyton, Lophophyton, Microsporum, Nannizia, Paraphyton, and Trichophyton* [1]. The commercial real-time multiplex PCR for the detection of dermatophytes directly from samples (skin, nail scrapings, and hair) was evaluated for its specificity and sensitivity. DermaGenius Nail, a commercial kit used in our two cases for identification of the fungus, was evaluated. The first retrospective evaluation of the kit from 138 fingernail and toenail specimens reported a sensitivity and specificity of the PCR test of 80% and 74.4%, respectively, when histology and culture were used as a reference to define onychomycosis [9].

Our two patients were treated with terbinafine. This molecule has shown its effectiveness on dermatophytes. Indeed, a study done by Badiee *et al*. which compared in vitro activities of new triazoles and classic antifungal agents against dermatophyte species isolated from Iranian university hospital, showed that luliconazole, terbinafine and isavuconazole in vitro revealed to be the most effective antifungal agents against all dermatophyte isolates [10].

## Conclusion

Conventional laboratory methods based on the detection of phenotypic features, such as microscopy and culture were the reference technique identification of dermatophytes. The morphological similarities between different species are observed. Novel molecular techniques have been developed for the rapid and accurate identification of dermatophytes.
